# Predictors of contraceptive use among adolescent girls and young women (AGYW) aged 15 to 24 years in South Africa: results from the 2012 national population-based household survey

**DOI:** 10.1186/s12905-019-0861-8

**Published:** 2019-12-12

**Authors:** L. Makola, L. Mlangeni, M. Mabaso, B. Chibi, Z. Sokhela, Z. Silimfe, L. Seutlwadi, D. Naidoo, S. Khumalo, A. Mncadi, K. Zuma

**Affiliations:** 1 Social Aspects of Public Health Research Programme, Human Sciences Research Council, Durban, South Africa; 2 Department of Psychology, University of Limpopo, Polokwane, South Africa; 3 Department of Psychology, University of KwaZulu-Natal, Durban, South Africa; 4 Critical Studies in Sexualities and Reproduction, Rhodes University, Makhanda, South Africa; 5 Human and Social Development, Human Sciences Research Council, Durban, South Africa

**Keywords:** Contraceptive use, Adolescent girls and young women, South Africa, Socio-demographic, Behavioral factors

## Abstract

**Background:**

Despite a variety of contraceptives being available for women in South Africa, a considerable number of adolescent girls and young women still face challenges in using them. This paper examines socio-demographic and behavioral predictors of using contraceptives among adolescent girls and young women (AGYW) aged 15 to 24 years.

**Methods:**

A secondary data analysis was conducted based on the 2012 population-based nationally representative multi-stage stratified cluster randomised household survey. Multivariate backward stepwise logistic regression model was used to examine socio-demographic and behavioural factors independently associated with contraceptive use amongst AGYW aged 15 to 24 years in South Africa.

**Results:**

Out of 1460 AGYW, 78% (CI: 73.9–81.7) reported using some form of contraceptives. In the model, contraceptive use was significantly associated with secondary education [OR = 1.8 (1.2–2.7), *p* = 0.005], having a sexual partner within 5 years of their age [OR = 1.8 (1.2–2.5), *p* = 0.002], and sexual debut at age 15 years and older [OR = 2.5 (1.3–4.6), *p* = 0.006]. The likelihood of association decreased with other race groups-White, Coloured, and Indians/Asians [OR = 0.5 (0.3–0.7), *p* = 0.001], being married [OR = 0.4 (0.2–0.7), *p* = 0.001], never given birth [OR = 0.7 (0.5–0.9), *p* = 0.045], coming from rural informal [OR = 0.5 (0.3–0.9), *p* = 0.010] and rural formal settlements [OR = 0.5 (0.3–0.9), *p* = 0.020].

**Conclusions:**

Evidence suggest that interventions should be tailor-made to meet the needs of AGYW in order to, promote use and access to contraceptives. The results also suggest that family planning interventions should target those who had not given birth in order to reduce unplanned and or unintended pregnancies and associated risk factors. These findings contribute to public health discourse and reproductive health planning for these age groups in the country.

## Background

The consistent and correct use of contraceptives does not only prevent unplanned and or unintended pregnancies but plays a significant role in reducing maternal morbidity and mortality amongst adolescent girls and young women (AGYW) [1]. It is envisaged that universal access and use of contraceptives amongst AGYW could lead to a decrease of 2.1 million unplanned and or unintended births, 3.2 million abortions, and 5600 maternal deaths each year [2]. Contraceptive use further provides AGYW with an opportunity to make informed decisions about when they want to have their children [3]. Despite these benefits, 214 million women from developing countries had an unmet need of modern contraceptives with approximately 53% Southern Africa women not using modern contraceptives in 2014 [4]. The 2016 South African Demographic and Health Key Indicators Report also shows that although 58% of South African women are in use of modern contraceptives, 18% of them still have unmet need for family planning. An unmet need occurs when a female in her reproductive age is married or unmarried, sexually active and fertile but is not using any method of contraception and does not want to have a child in the next two years or at all [5].

There are several factors predicting none or low usage of contraceptives amongst women. Research evidence has shown that some socio-demographic and behavioral factors influence contraceptive use [6–8]. For example, low contraceptive use has been associated with socio-demographic factors such as women coming from rural areas, those with low educational qualification, low socio-economic status, limited knowledge on accessibility, and awareness of contraceptives [9]. Previous findings also associated poor contraceptive usage with poor knowledge [10]. Furthermore, lack or low contraceptive use was associated with the fear of being labelled wayward and promiscuous for carrying or demanding usage of condoms by their partners [11].

Several behavioral and environmental factors have been suggested to affect correct usage, continuity and method of contraceptives preferred by AGYW. For instance, the age at sexual debut, coerced sexual intercourse, age disparate sexual relationships was associated with influencing contraceptive use [12, 13]. In spite of these factors, the South African government continues to be committed to improving usage of contraceptives. This is in line with global developmental agendas such as Family Planning 2020 and goal no 3.7 of the Sustainable Development Goal (SDG) [14]. In view of these developments and challenges, it is important to have an improved understanding of factors that influence contraceptive use amongst AGYW in order to strengthen current interventions. This paper examines socio-demographic and behavioral predictors of contraceptive use among adolescent girls and young women (AGYW) aged 15 to 24 years using data from the 2012 South African cross-sectional population-based household based survey.

## Methods

### Study design and sample

The analysis used data from the 2012 nationally representative population-based household survey conducted using a multistage stratified design described in detail elsewhere [10]. Briefly, a probability sample of 15 households drawn from 1000 randomly selected randomly selected enumeration areas (EAs) was used. The selection of EAs was stratified by province and locality defined as urban formal, urban informal, rural formal (including commercial farms), and rural informal (including tribal authority areas). In the formal urban areas, race was also used as an additional stratification variable unit. In each sampled household all persons residing in the household were invited to participate [15].

Age specific questionnaires were administered to consenting participants. Respondents were asked about background socio-demographic characteristics, HIV related knowledge, attitudes, and behaviours, reproductive history and contraceptive use. The current analysis focuses on the contraceptive use among a sub-sample of sexually active AGYW aged 15–24 years in relation to socio-demographic and behavioural characteristics. This paper only reports on the 1406 participants that answered questions regarding contraception use.

### Measures

#### Response variable

The primary outcome variable for this analysis is women’s utilization of any contraceptive method including female sterilization, male sterilization, the pill, the intrauterine device (IUD), injectables, rhythm (periodic abstinence) method, implants, male condom, female condom, withdrawal, emergency pill. The response variable was dichotomized based on a contraception method use question “are you or your partner currently using the following method to prevent pregnancy (yes/no) and the response was coded as 1 if individuals replied that they were using at least one of the above and 0 if they were not using any of the above.

#### Explanatory variables

Similar to the explanation provided in another study [16], the explanatory variables used were socio-demographic and behavioural factors. For characterisation of socio-demographics, age was grouped into two categories namely: 15 to 19 years and 20 to 24 years. Race was categorised as follows: Black African or other races - White, Coloured, and Indians or Asians. Similarly, marital status was categorised as not married or married, educational level (no education or primary school, secondary school, and tertiary education), employment status (not employed or employed), asset based socio-economic status score (a composite measure based on availability of essential services and ownership of a range of household assets), and locality type (urban formal, urban informal, rural informal, and rural formal).

Behavioural factors included age at sexual debut (younger than 15 or 15 years or older), number of sexual partners in the past year (1 partner, more than 1 partner), Alcohol use risk score (abstainers or low risk users, high risk or hazardous users) based on Alcohol Use Disorder Identification Test (AUDIT) scale, knowledge of HIV (no, yes), self-perceived risk of HIV infection (low, high), awareness of HIV status (yes, no), age of sexual partners (within 5 years, at least 5 years younger, at least 5 years older), ever tested for HIV (yes, no), ever given birth (yes, no), and HIV status (negative, positive).

### Statistical analysis

Descriptive statistics were used to summarize the data. Chi-square analysis was performed to test for differences in proportions of categorical variables. Multivariate backward stepwise logistic regression set at 0.1 was used to determine factors independently associated with contraceptive use. Odds ratios (OR) with 95% confidence intervals (CI) were used as measures of the direction and strength of the relationships, and a *p* ≤ 0.05 was considered statistically significant. All analyses were performed using STATA statistical software version 12.0 (Stata Corporation, College Station, USA).

## Results

### Sample characteristics and contraceptive use

Table 1 describes the study sub-sample and contraceptive use by socio-demographic profile of those individuals who reported sexual activity in the past year. Most participants were aged 20 to 24 years old (70.0%), were Black Africans (86.4%), not married (91.0%), had no education or had primary education only (54.6%)), were unemployed (81.9%), were from low SES households (46.6%), and lived in urban formal settlements (48.1%).

**Table 1:** Socio-demographic profile and contraceptive use among adolescent girls and young women (15–24 years)

Variables	Study Sample		Contraceptive use		*p*-value
	N	%	%	95% CI	
Age (years)					
15 to 19	424	30.0	53.7	46.4–60.9	0.117
20 to 24	977	70.0	60.6	55.2–65.8	
Race groups					
Black African	1023	86.4	60.2	55.2–64.9	0.038
Others	376	13.6	48.3	38.3–58.4	
Marital status					
Not Married	1277	91.0	57.9	53.5–62.2	0.434
Married	112	9.0	64.9	46.8–79.6	
Education level					
No education/Primary	724	54.6	62.2	56.2–67.8	0.245
Secondary	479	39.4	54.1	46.8–61.3	
Tertiary	63	6.0	60.0	39.8–77.2	
Employment status					
Unemployed	1088	81.9	58.8	54.1–63.4	0.988
Employed	248	18.1	58.7	47.0–69.6	
Asset based SES					
Low SES	571	46.6	66.7	60.1–72.7	0.001
Middle SES	551	33.1	57.1	50.4–63.5	
High SES	253	11.9	39.2	28.8–50.5	
Locality type					
Urban formal	685	48.1	54.7	47.9–61.2	0.124
urban informal	217	8.8	63.5	53.3–72.6	
rural informal	389	39.3	63.2	55.8–70.0	
rural formal	110	3.8	48.0	31.0–65.5	

*Not all sub-total add to the overall totals due to non-response and missing data, *SES* socio-economic status, *CI* confidence intervals

Table 1 also shows that there was a significant difference in the proportion of Black African (60.2%; 95% CI: 55.2–64.9) individuals that reported using contraceptives compared to other race groups (48.3%; 95% CI: 38.3–58.4; *p* = 0.038). They also show that there was a significant difference in the proportion of individuals who reported using contraceptives when comparing individuals from low SES households (66.7%; 95% CI: 60.1–72.7), middle SES households (57.1%; 95% CI: 50.4–63.5) and high SES households (39.2%; 95% CI: 28.8–50.5; *p* = 0.001).

Table 2 describes the study sample and contraceptive use by behavioural factors amongst AGYW who reported sexual activity in the previous year. Most of the participants had sexual debut at age 15 years or older (95.1%), had one sexual partners in the previous year (952.3%), had a sexual partner aged within 5 years of their age (65.1%), were abstainers/low risk alcohol users (90.3%), had never given birth (58.4%), had correct knowledge of HIV (50.7%), believed they were at a low risk of HIV infection (69.6%), and ever tested for HIV (82.3%).

**Table 2:** Behavioural factors and contraceptive use among adolescent girls and young women (15–24 years)

Variables	Study sample		Contraceptive use		
	N	%	%	95% CI	p-value
Age at sexual debut					
Younger than 15	77	4.9	61.2	45.1–75.3	0.755
15 years and older	1273	95.1	58.7	54.1–63.2	
Sexual partners in the last 12 months					
1 partner	1087	92.3	60.9	55.9–65.7	0.200
2+ partners	102	7.7	51.7	38.0–65.2	
Age of Sexual Partner					
Within 5 years	754	65.1	62.4	56.6–67.9	0.344
Five years younger	1	0	100		
Five years older	430	34.9	57.6	49.5–65.2	
Alcohol use risk score (AUDIT)					
Abstainers/low risk users	1115	90.3	59.7	54.9–64.2	0.387
High risk/hazardous users	110	9.7	50.8	31.6–69.7	
Ever Given Birth					
Yes	790	58.4	68.7	63.1–73.8	< 0.001
No	583	41.6	44.3	38.0–50.9	
Correct Knowledge of HIV					
No	702	49.3	60.7	54.5–66.6	0.306
Yes	693	50.7	56.3	50.2–62.3	
Self-perceived risk of HIV Infection					
Low	413	30.1	69.5	61.7–76.3	< 0.001
High	979	69.9	53.7	48.5–58.9	
Ever Tested for HIV					
Yes	1152	82.3	62.9	58.1–67.5	< 0.001
No	246	17.7	38.1	28.6–48.6	
Awareness of HIV status					
Yes	847	62.7	62.7	57.3–67.7	0.007
No	534	37.3	51.6	44.7–58.4	
HIV Status					
Negative	1013	83.5	59.1	53.9–64.1	0.570
Positive	171	16.5	62.3	51.7–71.8	

*Not all sub-total add to the overall totals due to non-response and missing data, *CI* confidence intervals, *AUDIT* Alcohol Use Disorder Identification Test

Table 2 also shows that there was a significant difference in the proportion of individuals who reported using contraceptives when comparing those who had given birth before (68.7%; 95% CI: 63.1–73.8) and those who had never given birth (44.3%; 95% CI: 38.0–50.9; *p* <  0.001). There was also a significant difference in the proportion of individuals who reported using contraceptives when comparing those who believed that they were at a low risk of HIV infection (69.5%; 95% CI: 61.7–76.3) and those who believed that they were at a high risk of HIV infection (53.7%; 95% CI: 48.5–58.9; p <  0.001). There was a significant difference in the proportion of individuals who reported using contraceptives when comparing those who reported that they had previously tested for HIV (62.9%; 95% CI: 58.1–67.5) and those who had never tested for HIV (38.1%; 95% CI: 28.6–48.6; p <  0.001). Finally, there was a significant difference in the proportion of individuals who reported using contraceptives when comparing those who were aware of their HIV status (62.7%; 95% CI: 57.3–67.7) and those who had never tested for HIV (51.6%; 95% CI: 44.7–58.4; *p* = 0.007).

### Types of contraceptives used

**Fig. 1:**
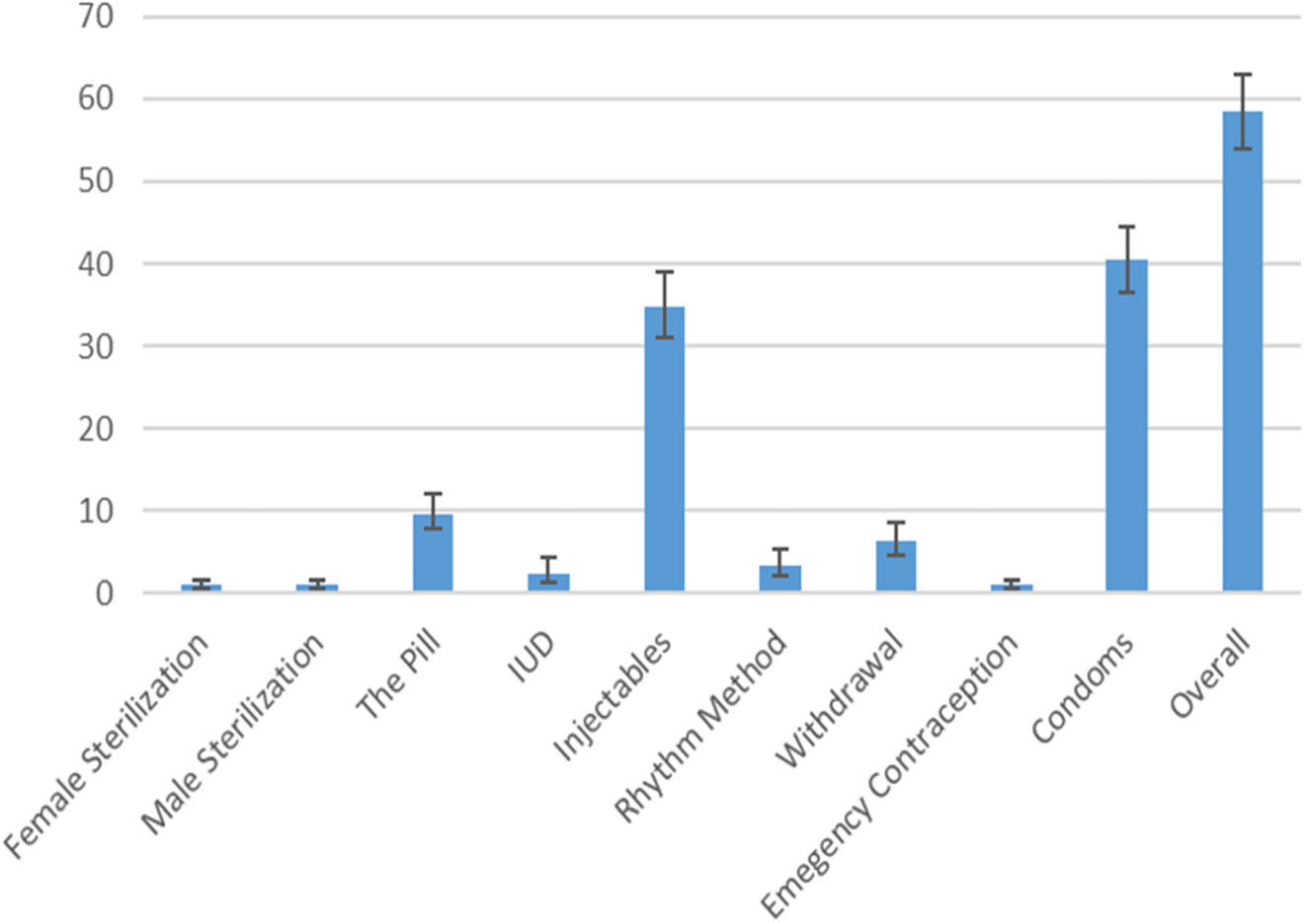
Types of contraceptives used by AGYW

Figure 1 below shows that 1% of women reported that they were sterilized, 1% of females reported that their partner was sterilized, 9.6% of females reported that they were on the pill, 2.3% reported that they had an intrauterine device, 34.8% reported that they were using injectables, 3.3% reported that they were using the rhythm method, 6.3% reported that they were using the withdrawal method, 1% reported that they used the emergency contraception, and 40.6% reported using condoms.

### The multivariate regression model

**Fig. 2:**
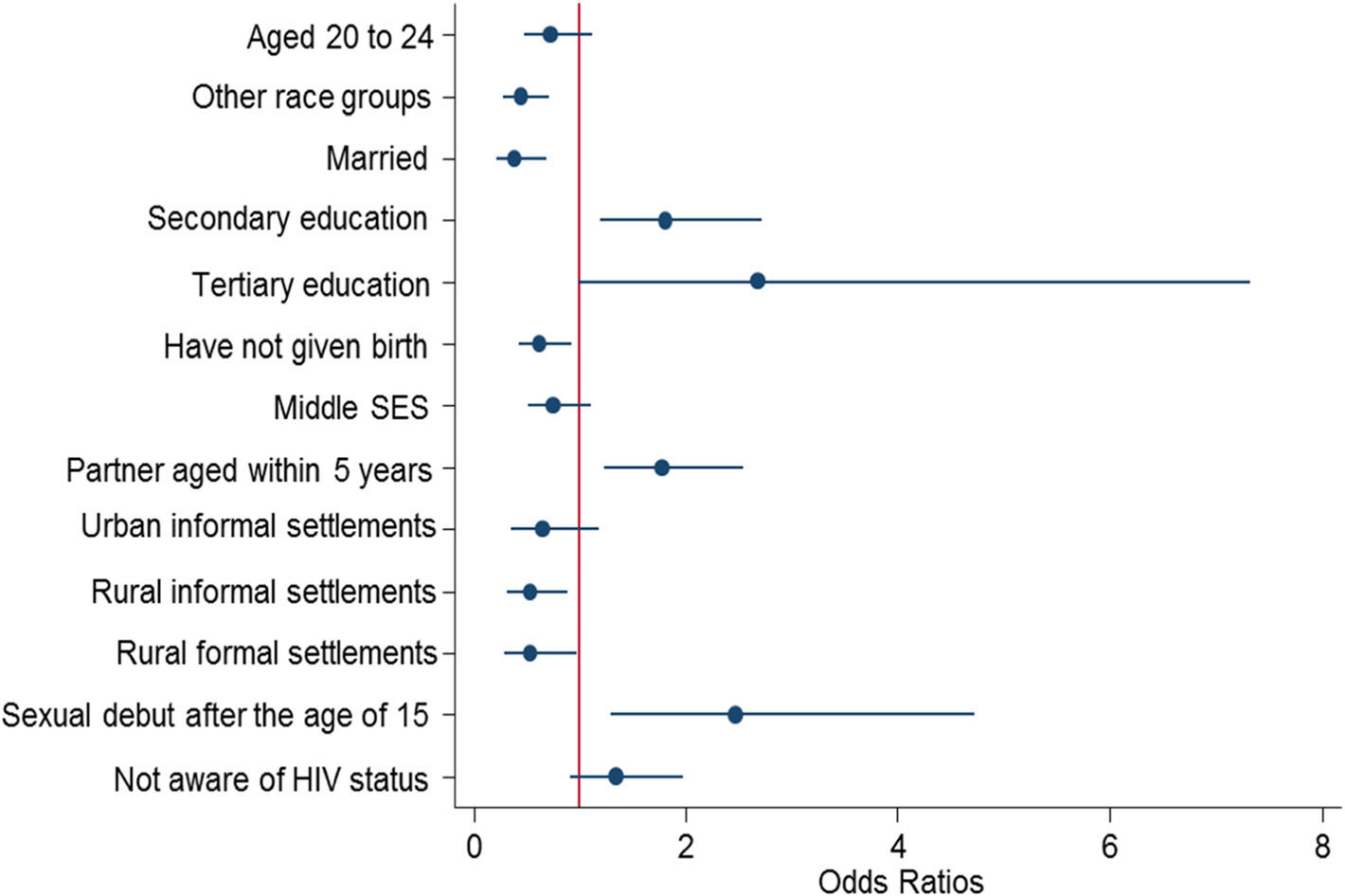
Multivariate model of factors associated with contraception use

The multivariate analysis (Fig. 2) shows that contraceptive use was significantly more likely among participants who had a secondary education compared to those with no education or primary level education [OR = 1.8 (95%CI: 1.2–2.7), *p* = 0.005]. The increased likelihood of using contraception was also partially significant among those with tertiary education [OR = 2.7 (95%CI: 0.9–7.3), *p* = 0.055]. In addition, participants with sexual partners within 5 years of their age were significantly more likely to use contraceptives than those who had sexual partners that were 5 years older than them [OR = 1.8 (95%CI: 1.2–2.5), *p* = 0.002]. Participants who had their sexual debut at age 15 years or older were significantly more likely to use contraceptives than those who had their sexual debut before the age of 15 years [OR = 2.5 (95%CI: 1.3–4.6), *p* = .006].

Furthermore, participants of other race groups were significantly less likely to use contraceptives when compared to Black Africans [OR = 0.5 (95%CI: 0.3–0.7), *p* = 0.001]. Married individuals were significantly less likely to use contraceptives when compared to their unmarried counterparts [OR = 0.4 (95%CI: 0.2–0.7), *p* = 0.001]. Participants that had never given birth were significantly less likely to use contraceptives compared to participants who had ever given birth [OR = 0.7 (95%CI: 0.5–0.9), *p* = 0.045]. Contraceptive use was also significantly less likely among participants from rural informal [OR = 0.5 (95%CI: 0.3–0.9), *p* = 0.010] and rural formal settlements [OR = 0.5 (95%CI: 0.3–0.9), *p* = 0.020] compared to those from urban formal settlements.

## Discussion

The findings of this nationally representative survey suggest that there are socio-demographic and behavioral factors influencing the use of contraceptives. For socio-demographic factors, important predictors of contraceptive use among AGYW were educational level, locality type and marital status. Similarly, other studies also found that contraceptive use was higher among young women with secondary and tertiary education compared to those with primary education [9, 17]. Education exposes women to reproductive health information and empowers them to make informed decisions about reproductive health and methods available for them [18]. It is important for policy makers to develop interventions targeting on the differences in the subgroups of women.

In line with the current findings, other studies also found that young women from rural informal and formal settlements were less likely to use contraception compared to those from urban formal settlements [19, 20]. This has been attributed to limited awareness of contraceptive methods available to them and challenges with access to health care services [21]. It is particularly concerning that family planning remains a challenge to many rural AGYW in the country despite policy and interventions aimed at improving sexual and reproductive health information [22]. It is therefore important to improve accessibility to contraceptives through improving awareness on the types of contraceptives available, benefits of using them and access of contraceptives. The relationship between nursing staff at the clinics and young girls should also be friendly and non-judgemental environment. Stereotypes towards contraceptives should be addressed in order to encourage use.

Also amongst socio-demographic factors, marriage was also associated with non-use of contraceptive. This finding was previously linked to the desire to have children within marriage, cultural norms or opposition by the husbands [23, 24, 7]. This finding could also suggest that the majority of unmarried AGYW are using contraceptives. This has positive implications since prevention of unintended pregnancy is considered a priority among policymakers and the public because of its high economic, social and health costs for young people and their families [25, 26].

Furthermore, among behavioral factors the results showed that important predictors of contraceptives use among AGYW were age at sexual debut, partner age difference and child bearing. The findings imply low contraceptive use amongst those who had sexual debut before the age of 15 years. Similarly, a previous study supports this finding [27]. The lack of contraceptive use amongst those who had sex before age 15 could be linked to lower knowledge and fear to access contraceptives at their age due to societal stigma. In addition, the perception that being exposed to contraceptives at an early age encourages adolescents to engage in sexual activities may be a hindrance to early initiation to contraceptives by both adolescents and parents [20, 28]. Others have shown that sexual debut at 14 year and younger without use of contraceptives was a strong predictors of the likelihood of inconsistent usage of contraceptives even in later sexual engagements [20].

The findings further suggest that having a sexual partner 5 years and older is associated with low contraceptive use compared to having a sexual partner within one’s age. Epidemiologic studies of relationships and contraceptive use have demonstrated a strong association between relationship type, and contraceptive decision-making including condom use [26]. For example, evidence shows that condom use is negotiable when the age range between partners is within 5 years, and this has been linked to gender-power dynamics [26, 29–32]. The results also showed that contraceptive use was less likely among AGYW who had not given birth as observed in other studies [33]. Family planning interventions should be tailored to target these specific groups of women.

### Limitations

This study has some limitations and should be should also be interpreted with caution. First, the data is based on cross-sectional design and, therefore cannot be used to infer causality. In addition, the analysis was based on self-reported information that may be influenced by recall and social desirability bias. Nevertheless, this paper provides an insight into the predictors of contraceptive usage amongst adolescent girls and young South African women (15–24 years) using nationally representative data. Therefore, the findings of this study contributes to public health discourse and reproductive health planning for AGYW.

## Conclusion

The findings highlights a need to promote the availability and acceptability of the different contraceptives for use by AGYW. There is need to improve awareness on family planning services and increase access to contraceptives where needed. It further calls for the reinforcement of a non-judgmental environment in health facilities. Interventions should be tailor made and incorporate peer-mediated interventions because of the differences in women according to their educational level, place of residence, and marital status. There is also a need for age specific message to prevent and or delay early sexual debut and unplanned pregnancies until adolescents and young women have sufficient information and can thus make informed decision regarding their sexuality and fertility. Partners especially males have been associated with low usage of contraceptives amongst women. This is a clear indication that barrier methods is not just a ‘woman problem’ and although she often bares the consequences of an unplanned pregnancy, their male partners have significant influence in whether they use contraceptives or not. Thus, not only do policy makers and interventions need peer-mediated and fit-for-purpose interventions, it is critical that males are made part of the discourse and solution for the utilization of contraceptive methods.

## Data Availability

The dataset(s) could be available through the Human Sciences Research Council data research repository via access dataset http://www.hsrc.ac.za/en/research-data/ upon request.

## Abbreviation

AGYW: Adolescent girls and young women
